# Differentially Methylated Regions of Imprinted Genes in Prenatal, Perinatal and Postnatal Human Tissues

**DOI:** 10.1371/journal.pone.0040924

**Published:** 2012-07-13

**Authors:** Susan K. Murphy, Zhiqing Huang, Cathrine Hoyo

**Affiliations:** Department of Obstetrics and Gynecology, Duke University Medical Center, Durham, North Carolina, United States of America; University of Bonn, Institut of Experimental Hematology and Transfusion Medicine, Germany

## Abstract

Epigenetic plasticity in relation to *in utero* exposures may mechanistically explain observed differences in the likelihood of developing common complex diseases including hypertension, diabetes and cardiovascular disease through the cumulative effects of subtle alterations in gene expression. Imprinted genes are essential mediators of growth and development and are characterized by differentially methylated regulatory regions (DMRs) that carry parental allele-specific methylation profiles. This theoretical 50% level of methylation provides a baseline from which endogenously- or exogenously-induced deviations in methylation can be detected. We quantified DNA methylation at imprinted gene DMRs in a large panel of human conceptal tissues, in matched buccal cell specimens collected at birth and at one year of age, and in the major cell fractions of umbilical cord blood to assess the stability of methylation at these regions. DNA methylation was measured using validated pyrosequencing assays at seven DMRs regulating the *IGF2/H19*, *DLK1/MEG3, MEST, NNAT* and *SGCE/PEG10* imprinted domains. DMR methylation did not significantly differ for the *H19*, *MEST* and *SGCE/PEG10* DMRs across all conceptal tissues analyzed (ANOVA p>0.10). Methylation differences at several DMRs were observed in tissues from brain (*IGF2* and *MEG3-IG* DMRs), liver (*IGF2* and *MEG3* DMRs) and placenta (both *DLK1/MEG3* DMRs and *NNAT* DMR). In most infants, methylation profiles in buccal cells at birth and at one year of age were comparable, as was methylation in the major cell fractions of umbilical cord blood. Several infants showed temporal deviations in methylation at multiple DMRs**.** Similarity of inter-individual and intra-individual methylation at some, but not all of the DMRs analyzed supports the possibility that methylation of these regions can serve as useful biosensors of exposure.

## Introduction

The early origins hypothesis, popularized by Barker [1], postulates that the risk of developing complex diseases and disorders is established as an adaptive response to the perceived *in utero* environment. Compelling epidemiologic data in support of the early origins hypothesis derive from studies of individuals exposed to famine conditions in 1944–45 at the end of World War II [2] and those enduring the Chinese Famine of 1959–61 [3]. Individuals exposed to severe caloric restriction *in utero* have a higher incidence of type 2 diabetes [4], [5], coronary heart disease [6], schizophrenia [7], [8], [9], obesity [10], [11] and breast cancer [12], [13], [14] compared to those not exposed. In addition to these well-documented human disasters, recent studies on prenatal exposure to cigarette smoking show an increased risk of benign breast disease [15], and ADHD [16], [17]. Epigenetic mechanisms have been proposed to mediate these associations, supported by studies in mice that link maternal diet and exposures to phenotypic changes in the pups that are directly mediated by DNA methylation at particular loci [18], [19], [20], [21]. However, the identity of such epigenetic targets in humans remains largely unknown. Understanding the etiology of common chronic diseases will require a concerted effort to identify intermediate endpoints that can serve as a compendium of an individual’s prior exposure history.

Studies in humans present a substantial challenge to compiling such a compendium. While rodent models provide the means to address mechanistic questions in an isogenic background under carefully controlled conditions, the relevance of these same questions in humans is difficult to directly infer without epidemiological observation. Further complicating interpretation of such studies is the lack of tools, analogous to genotype, that provide an archival history of exposure. A growing body of evidence suggests that epigenetic features of the genome, meaning regulatory mechanisms that bring about changes in phenotype without changing the nucleotide sequence, provide a means by which past exposures can be ‘recorded’ [22]. As such, these features can be exploited to improve exposure assessment [23], [24]. DNA methylation is perhaps the most intensively studied epigenetic mechanism owing to its mitotic stability and the technologies available for quantifying measurement. However, the use and interpretation of DNA methylation profiles as relevant archives or biosensors in large population studies will require background knowledge of the significance of differences in methylation as well as the temporal stability of methylation marks. Epidemiologic investigations are limited to studies of readily available tissues, most often, peripheral blood and buccal cells. Thus, to be a useful epigenetic biosensor of early exposure, methylation patterns should be established prior to gastrulation and thus be systemically similar, exhibit stability over time, but also exhibit measurable variability in response to exposures.

Several genes have been shown to exhibit epigenetic responses to the environment, including those not subject to genomic imprinting [25], [26]. The well-characterized regulatory regions associated with genomically imprinted genes may provide a relatively convenient mechanism to detect methylation changes resulting from early exposures [27]. Imprinted genes exhibit expression from only one of the two parental alleles in a manner that depends on the parental origin of the allele. This is regulated by DNA methylation that is established differentially during gametogenesis such that these differentially methylated regions (DMRs) theoretically exhibit 50% methylation in diploid somatic cells. Because DMR methylation is remodeled and then firmly entrenched prior to germ layer specification, this methylation pattern is faithfully transmitted to daughter cells during somatic cell division and is therefore thought to be perpetuated throughout life in all tissues. Environmental influences that shift the fidelity of imprinted gene DMR reprogramming and maintenance have been documented, including for example *in utero* exposure to dietary micronutrients [28], [29], caloric restriction [25], [26], [30], protein restriction [31] and cigarette smoking [32]. Thus this particular group of genes may offer an opportunity to provide an accessible historical archive of such events [23], [24]. Herein we report on the methylation profiles of multiple imprinted gene DMRs across human tissue types and the stability of these marks during the prenatal and post-natal periods.

## Methods

### Ethics Statement

Participants were recruited after appropriate human subjects approvals were obtained and written informed consent was provided. All study protocols were approved by the Duke University Institutional Review Board.

### Specimens

Biological specimens analyzed included umbilical cord blood and buccal cells taken at birth and at one year of age from a subset of babies born to women consented as part of the Newborn Epigenetics STudy (NEST), a cohort study of newborns and their mothers, who were recruited when pregnant from prenatal clinics that serve Durham Regional Hospital or Duke Obstetrics, the two obstetrics facilities serving the County of Durham, NC. Gestational age at recruitment ranged from 19 to 42 weeks. Eligibility criteria included: 18 years and older, English speaking, seeking care at prenatal clinics serving the obstetrics hospitals and plans to deliver at Duke Obstetrics or Durham Regional Hospital. The details of the study population have been described elsewhere [33]. In addition, human conceptal tissues from elective pregnancy termination procedures, including adrenal gland, brain, eye, gonad, heart, intestine, kidney, liver, lung, muscle, pancreas, spleen and thymus, as well as placenta, umbilical cord and maternal uterine decidua were analyzed from up to 16 individuals. The gestational age distribution and sex were as follows: females, 57d, 80d, 87d, 94d, 101d, 105d, 108d (2 individuals), 120d and 122d; males, 58d, 80d, 98d, 113d, 120d and 125d (also see Table S1). Conceptal tissues were provided by the Laboratory of Developmental Biology at the University of Washington.

Umbilical cord blood (UCB) was collected via umbilical vein puncture into ethylenediaminetetraacetic acid-containing vacutainer tubes, inverted and centrifuged to harvest plasma and the leukocyte-containing buffy coat followed by storage at −80°C. Buccal cells were collected from infants at birth by trained research staff and again at one year of age from the same infants by trained study staff or by mothers receiving a mail kit containing questionnaire materials along with the Dacron swabs, instructions for collection and a self-addressed, postage-paid envelope for return to the study office and laboratory.

### Nucleic Acid Preparation

Genomic DNA was extracted from tissues using Puregene Reagents (Qiagen; Valencia, CA). UCB leukocyte DNA was purified using the Qiagen QIAamp DNA Mini kit and from buccal cells using the QIAamp DNA Investigator kit on a QiaCube instrument (Qiagen, Valencia, CA). DNA quality was assessed using a Nanodrop 1000 Spectrophotometer (Thermo Scientific; Wilmington, DE).

### DNA Methylation Analysis

We developed bisulfite pyrosequencing assays to quantitatively measure the level of methylation at CpG sites contained within seven regions of known differential methylation in human tissues. The seven DMRs analyzed include two involved in regulating the *DLK1/MEG3* imprinted domain on chromosome 14q32.2 (the *MEG3*-IG DMR and the *MEG3* DMR), one at the *MEST* promoter at 7q32.2, one at the *SGCE/PEG10* promoter region on 7q21.3, one at the *NNAT* locus at 20q11.23, and two that are involved in imprinting of the *IGF2/H19* domain on chromosome 11p15.5 (the *IGF2* DMR and the *H19* DMR) which are located upstream of the imprinted promoters of *IGF2* and at the imprinting control region for the *IGF2/H19* imprinted domain near the *H19* promoter, respectively [34]. Genomic DNA (500–800 ng) was treated with sodium bisulfite using the EZ DNA Methylation Kit per the manufacturer’s instructions (Zymo Research; Irvine, CA) to convert unmethylated cytosines to uracils, leaving methylated cytosines unchanged. Bisulfite converted DNA (∼20 ng) was amplified by PCR in a 25 µl reaction volume using the PyroMark PCR Kit (Qiagen) with 1.5 mM MgCl_2_ and 0.12 µM each of the forward and reverse PCR primers. Each reaction also contained 2.5 µl of CoralLoad Concentrate (Qiagen) for checking amplicons on an agorase gel. One primer of each primer pair was conjugated to biotin at the 5′ end in order to facilitate retention of a single strand using streptavidin beads for the pyrosequencing reaction. These single stranded amplicons were isolated using the Pyrosequencing Work Station and underwent pyrosequencing on a Pyromark Q96 MD pyrosequencing instrument (Qiagen). PCR and pyrosequencing primers, genomic coordinates and PCR amplification conditions are provided in Table 1. Pyrosequencing assays were performed in duplicate in sequential runs (technical replicates), and the values shown represent the mean methylation for the CpG sites contained within the sequence analyzed. Pyrosequencing assays (except for the *IGF2* and *H19* DMR) were validated using defined mixtures of Epitect control fully methylated and unmethylated human genomic DNAs (Qiagen). Validation of pyrosequencing assays for the *IGF2* and *H19* DMRs and analysis of 5% incremental increases in methylated DNA used defined mixtures of purified plasmid DNAs that contain the bisulfite modified version of the fully methylated or fully unmethylated target sequences [35].

**Table 1:** PCR and pyrosequencing primers (listed 5′ to 3′), genomic coordinates^1^ and reaction conditions.

DMR (Chr)	Forward primer	Reverse primer	Sequencing primer	PCR conditions
*H19* (11p15.5)	TTTGTTGATTTTATTAAGGGAG 2,011,131-2,011,153	*CTATAAATAAACCCCAACCAAAC2,011,253-2,011,275	GTGTGGAATTAGAAGT 2,011,192-2,011,207	95°C 15 min
				94°C−65°C−72°C×5 |30 s
				94°C−62°C−72°C×5 |30 s
				94°C−59°C−72°C×50 |30 s
				72°C 10 min; 4°C ∞
*IGF2* (11p15.5)	GGAGGGGGTTTATTTTTTTAGGAAG 2,151,629-2,151,653	*AACCCCAACAAAAACCACTAAACAC2,151,697-2,151,721	GGGGTTTATTTTTTTAGGA 2,151,633-2,151,651	5°C 15 min
				94°C−68°C−72°C×5 |30 s
				94°C−66°C−72°C×50 |30 s
				72°C 10 min
				4°C ∞
*MEG3* (14q32.2)	GGGATTTTTGTTTTTTTTTGTAGTAGG 101,294,220-101,294,246	*CCAACCAAAACCCACCTATAAC101,294,370-101,294,391	TTTGGGGTTGGGGTT 101,294,301-101,294,315	95°C 15 min
				94°C |30 s
				60°C |30 s×55
				72°C |30 s
				72°C 10 min; 4°C ∞
*MEG3-*IG (14q32.2)	TTGGAATTGTTAAGAGTTTGTGGATT 101,277,178-101,277,203	*AATTAACAAACCATAAACAACTATAAACC101,277,377-101,277,405	GGATTTGTGAGAAATGAT 101,277,199-101,277,216	95°C 15 min
				94°C |30 s
				61°C |30 s×55
				72°C |30 s
				72°C 10 min; 4°C ∞
*MEST* (7q32.2)	GGTGAGATTAGGGTTATTATGGAT 130,132,476-130,132,499	*AAAAAAAAATATCACTCCTACCC130,132,339-130,132,361	GAAATTTTAAATTTTATTA 130,132,425-130,132,443	95°C 15 min
				94°C |30 s
				63°C |30 s×55
				72°C |30 s
				72°C 10 min; 4°C ∞
*PLAGL1* (6q24.2)	GTAGGGTAGGTGTTTGGGTGTT 144,329,210-144,329,231	*CRACAAAAACACACCCTCCTC144,329,109-144,329,129	GTAGGTGTTTGGGTGTT 144,329,210-144,329,226	5°C 15 min
				94°C |30 s
				68°C |30 s×55
				72°C |30 s
				72°C 10 min; 4°C ∞
*PEG10* (7q21.3)	*GTGTTAAGGAGTTGGGAGGA 94,217,815-94,217,771	TCTACAACCCTATAACAACCAATCTCA94,217,907-94,217,933	CCTAATATACCTTCTCTA 94,217,873-94,217,890	95°C 15 min
				94°C |30 s
				64°C |30 s×55
				72°C |30 s
				72°C 10 min; 4°C ∞
*NNAT* (20q11.23)	TAAATTTGTAGGTTAGGGATTGGG 36,169,325-36,149,348	*CCAAAAAAAAAAAAAAATAATCCATCTACT36,149,515-36,149,544	TTGTAGGTTAGGGATTG 36,149,330-36,149,346	95°C 15 min
				94°C |30 s
				63°C |30 s×55
				72°C |30 s
				72°C 10 min;4°C ∞

* Biotin tagged primer.

1 Genomic coordinates based on UCSC Genome Browser, February 2009 release, GRCh37/hg19.

### Fractionation of Umbilical Cord Blood

Specimens were collected into bags containing acid citrate dextrose anticoagulant and subsequently fractionated using Lympholyte®-poly (Cedarlane Laboratories Limited, Burlington, NC, USA) to separate and collect the polymorphonuclear (PMN) and peripheral blood mononuclear cell (PBMC) fractions. Purity of the PMN and PBMC fractions was examined following Giemsa staining. Cells were examined using a 63× oil immersion lens on a Zeiss Axiovert 25CFL inverted microscope with a 10× objective. Digital micrographs were taken using a Zeiss Axiocam MRm camera.

### Statistical Analyses

The relationship between methylation levels in paired tissues (umbilical cord blood versus buccal cells at birth, and buccal cells at birth and buccal cells taken at one year of age) was assessed using nonparametric paired t tests. Cross-tissue comparisons of methylation at each DMR were evaluated using one-way analysis of variance (ANOVA), to compare continuous variable measurements among three or more groups. This analysis involved using multiple available tissues for each individual but the available tissues were not identical between individuals. Thus all available data for each individual was included in this analysis (see Table S1), with the p value shown indicating if there was a significant difference in methylation across tissues. Universally methylated controls (UMD) were excluded from the ANOVA comparisons, as were tissues where only one specimen was analyzed. Where ANOVA p values were significant, a Bonferroni multiple comparisons post test correction was used to determine which tissues were driving the significant p value (designated by the lighter grey bars in the figure); exclusion of these tissues from the ANOVA comparison where relevant resulted in a non-significant p value (not shown). The relationship between gestational age and methylation was evaluated using Spearman correlation. Comparisons of methylation values present in fractionated umbilical cord specimens were done using paired t tests. P values <0.05 were considered significant.

## Results

### Assay Validation

We developed bisulfite pyrosequencing assays to quantitatively measure the level of methylation at CpG sites contained within seven regions of known differential methylation in human tissues. Details of the pyrosequencing assays, including primers used, are provided in Table 1. PCR optimization was followed by pyrosequencing in duplicate to test the linearity in measurements of increasing amounts of input methylated DNA, using defined mixtures of commercially available methylated and unmethylated DNAs. The seven DMRs analyzed include two that are involved in imprinting of the *IGF2/H19* domain on chromosome 11p15.5 (the *IGF2* DMR and the *H19* DMR, both paternally methylated; *IGF2*, OMIM:147470; *H19,* OMIM:103280), two involved in regulation of the *DLK1/MEG3* imprinted domain on chromosome 14q32.2 (the *MEG3*-IG DMR and the *MEG3* DMR, both paternally methylated; *DLK1*, OMIM:176290; *MEG3*, OMIM:605636), one at the maternally methylated *MEST* promoter region at 7q32.2 (OMIM:601029), one at the maternally methylated *SGCE/PEG10* promoter region on 7q21.3 (*SGCE,* OMIM:604149; *PEG10*, OMIM:609810), and one at the maternally methylated *NNAT* locus at 20q11.23 (OMIM:603106).

**Figure 1:**
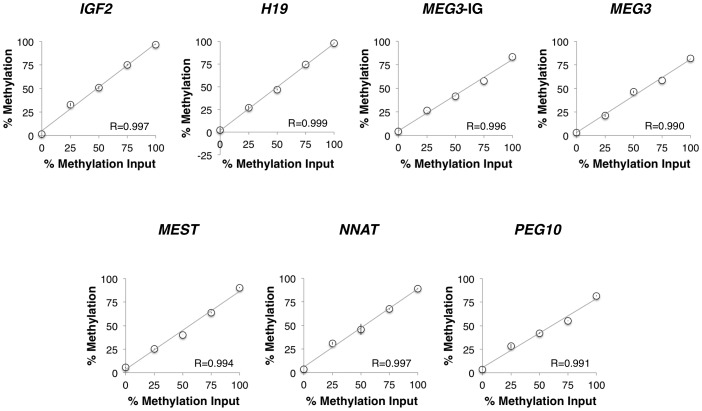
Pyrosequencing validation using whole genome-amplified and bisulfite modified mixtures of fully methylated and unmethylated DNAs. The percent of methylated DNA in each specimen analyzed is shown on the x-axis while the actual percent of methylation measured by pyrosequencing is shown on the y-axis. Error bars indicate the standard deviation for duplicate or triplicate measures.

The *IGF2* and *H19* DMRs were validated using mixtures of plasmids containing the bisulfite modified versions of the fully methylated or fully unmethylated sequences, as described previously [35]. Because the plasmids can be prepared in large quantities, this technique allows for better precision in quantifying the DNA and thus in preparing mixtures with defined methylated:unmethylated ratios. This also improves the ability to detect a fully methylayed template as compared to using DNA treated with *SssI* methyltransferase, which often shows less than complete methylation (see below). As shown in Figure 1, the degree of methylation measured matched that of the methylation input across the range of values measured for both of these DMRs (Pearson r = 0.997 for the *IGF2* DMR and r = 0.999 for the *H19* DMR), showing a linear increase with no apparent bias in amplification.

For the other DMRs, we analyzed mixtures of commercially available bisulfite modified methylated and unmethylated DNAs containing 0%, 25% 50% 75% and 100% methylated DNA. The measured level of methylation was lower than that expected for all but the 0% methylated DNA, likely due to incomplete enzymatic methylation of the DNA by the *Sss*1 methyltransferase as has been reported previously [36], [37]. Regardless, as was observed for the *IGF2* and *H19* DMRs, each increase in the amount of input methylated DNA was accompanied by a proportional increase in the amount of methylation measured, with Pearson r values between 0.990 and 0.997 (Figure 1).

**Figure 2:**
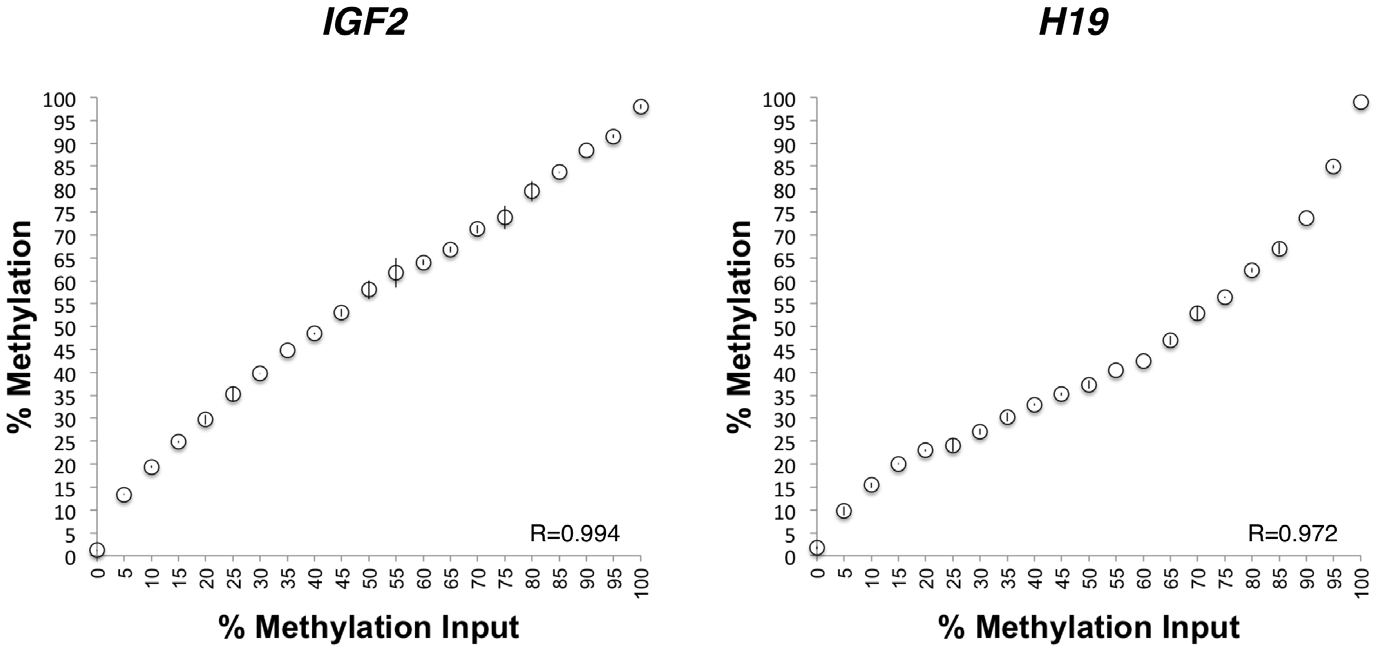
Pyrosequencing validation using bisulfite modified methylated and unmethylated *IGF2* and *H19* DMR sequences in plasmids. Plasmids were quantified and mixed by pipetting to generate specimens containing 5% incremental increases in methylated DNA over the full range of possible methylation values (0% to 100%). Error bars, SD for duplicate measurements.

### Detectable Methylation Differences

One question not previously addressed, to our knowledge, is the limit of differential methylation detectable by pyrosequencing. This is critically important for studies in which small differences in methylation may be observed between individuals or groups, as is expected for methylation shifts resulting from adaptations to environmental conditions [30] as opposed to the more substantial epigenetic changes found in pathological conditions like cancer [34]. To address this, we prepared mixtures of the plasmid DNAs containing the methylated and unmethylated versions of the bisulfite modified sequence for the *IGF2* and *H19* DMRs, described above. We hand-pipetted mixtures from 0% to 100% methylated DNA in 5% increments, and measured methylation in duplicate by pyrosequencing. Each of these DMRs showed corresponding increases in measured methylation with each 5% increase in input methylated DNA, with Pearson rho values of 0.994 and 0.972 for the *IGF2* and *H19* DMRs, respectively (Figure 2). The higher measured levels of methylation at the *IGF2* DMR relative to input is likely due to underestimation of the actual amount of methylated DNA in the stock used for preparation of these dilutions, especially since the mixtures containing a higher proportion of methylated DNA had actual measurements that more closely matched the anticipated values. Results for the *H19* DMR show measured methylation less than that expected at levels above ∼20% input methylation, again suggesting that this may be due to underestimation of the unmethylated template concentration rather than amplification bias, especially since the data shown in Figure 1 for this DMR indicate lack of bias using the same assay and template, differing only in the preparation of the mixtures. These results unequivocally demonstrate that methylation differences as low as 5% are distinguishable by bisulfite pyrosequencing across the full dynamic range of the assay.

### Imprinted Gene DMR Profiles in Human Conceptal Tissues

**Figure 3:**
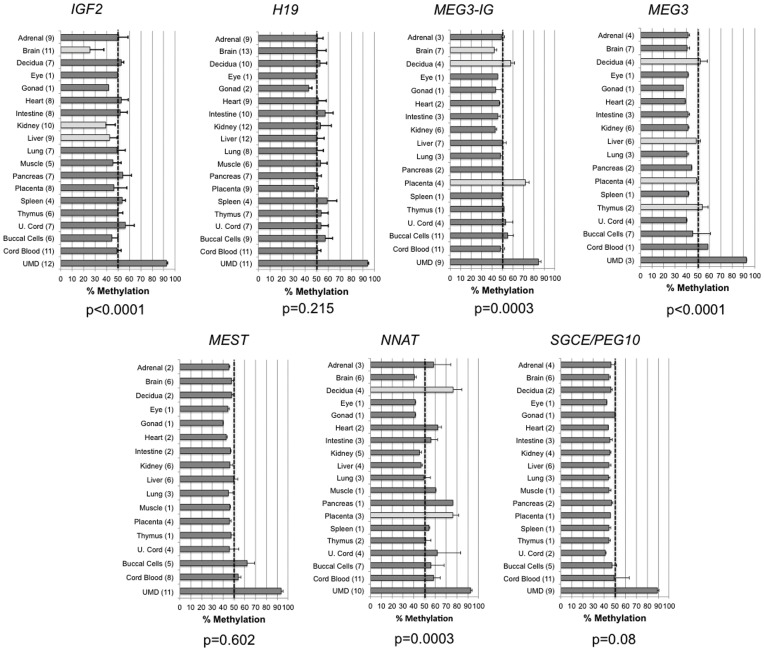
Methylation of seven imprinted gene DMRs across a wide range of human tissues. The number of specimens analyzed is indicated within the parentheses after each tissue listed on the vertical axes. UMD, universally methylated DNA; error bars, SD across tissues; where only one tissue was analyzed, error bars represent the SD for replicate measures. ANOVA analysis (excluding UMD) p values are shown below each graph. Light grey bars designate those tissues showing deviation in methylation from the average; when removed, the p value becomes non-significant (i.e., p≥0.05).

Figure 3 shows DMR methylation levels from tissues derived from each of the three germ layers in up to fourteen individual human conceptuses, gestational ages ranging from 58–125 days, including ectoderm (buccal cells, brain and eyes), endoderm (adrenal medulla, intestine, liver, lung, pancreas and thymus) and mesoderm (gonads, heart, kidney, muscle, spleen and umbilical cord blood). We also included tissues specific to pregnancy from these tissues, including decidua, placenta and umbilical cord. Methylation levels were similar for the *H19*, *MEST* and *SGCE/PEG10* DMRs across all conceptal tissues analyzed (p = 0.215, p = 0.874 and p = 0.197, respectively). For the other DMRs, methylation profiles were largely consistent across tissues, but there were some differences noted. For example, at the *IGF2* DMR, brain, kidney and liver showed lower overall average methylation than the other tissues analyzed, while the remaining tissues show no significant difference in methylation. At the *MEG3-IG*, *MEG3* and *NNAT* DMRs, methylation was higher in pregnancy-related tissues relative to the other organ tissues analyzed. Higher methylation in these tissues may reflect a strong requirement to control imprinting and/or expression levels, as these specialized tissues control the allocation of nutrients and oxygen to the developing fetus and imprinted genes are known to have particularly important roles in this process [38].

**Figure 4:**
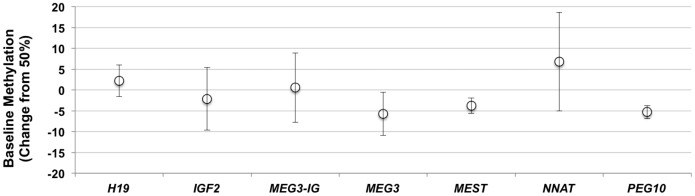
Deviation of methylation levels from 50% baseline across imprinted DMRs. Values shown represent the average of all methylation values obtained across all tissues analyzed in Figure 3. The theoretical 50% level of methylation anticipated is represented as the baseline (0). Error bars, SD for all tissues analyzed.

The theoretical baseline level of 50% methylation at imprinted DMRs, as described above, was close to that detected across the tissues examined when averaged for all specimens analyzed (Figure 4). The lowest average level of methylation was detected at the *MEG3* and *SGCE/PEG10* DMRs (44.3%, SD = 5.2% and 44.7%, SD = 1.6%, respectively), while the *NNAT* DMR showed the highest average and most variable levels of methylation across tissues (56.8%, SD = 11.9%). The *H19*, *MEST* and *SGCE/PEG10* DMRs showed the least variation in methylation (SD = 3.8%, 1.9% and 1.6%, respectively).

**Figure 5:**
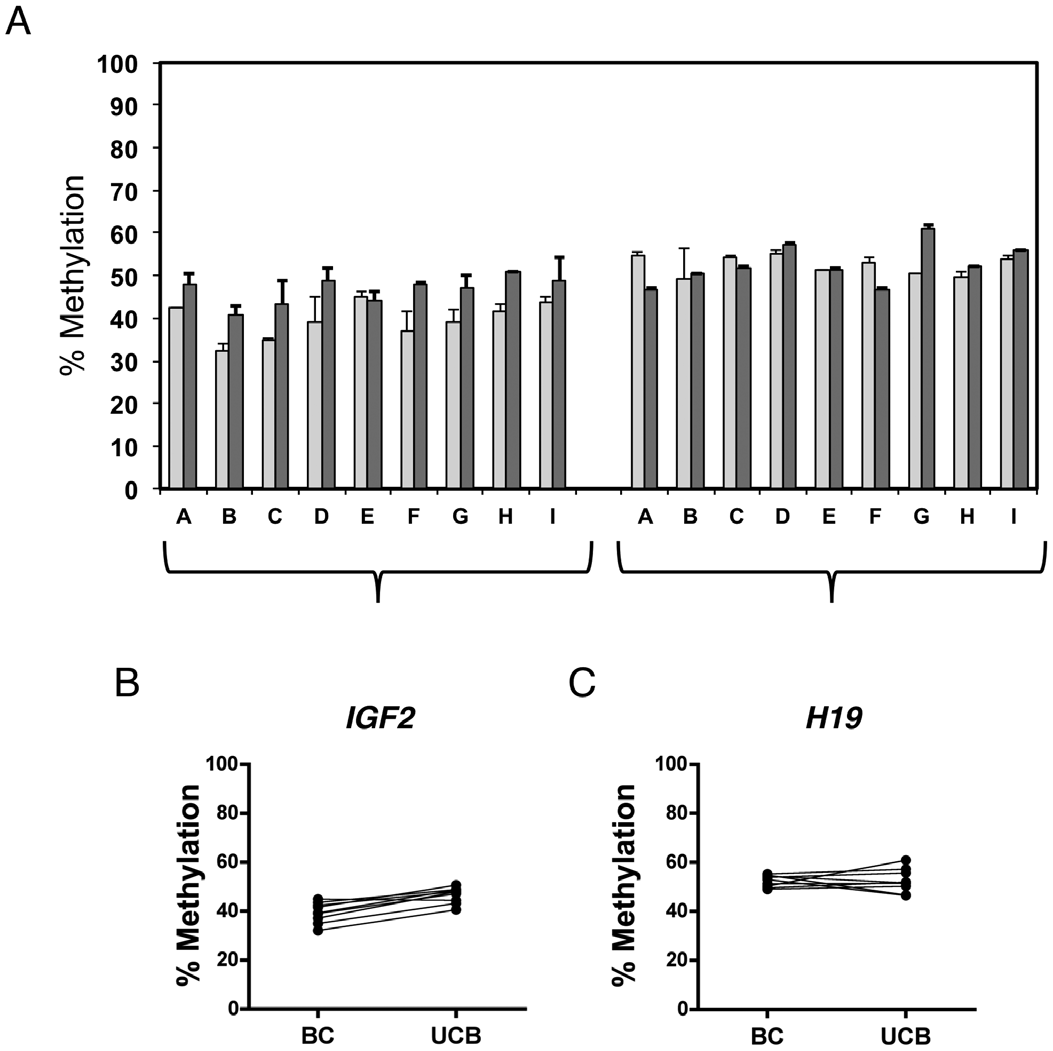
Matched umbilical cord blood and buccal cell methylation profiles. (A) Analysis of methylation at the *IGF2* and *H19* DMRs in 9 pairs of matched buccal cells (BC; light grey bars) collected at birth and umbilical cord blood (UCB; dark grey bars). (B) and (C) show a direct comparison between these tissue types for the same specimens. *IGF2* DMR, R = 0.61, (p = 0.08); *H19* DMR, R = −0.10 (p = 0.81).

We also analyzed the relationship between gestational age and methylation in these tissues and found no significant correlations, with the sole exception of *MEG3-IG* in brain (Table S2). In this case, the methylation levels are strongly positively correlated with gestational age, and ranged from 39.1% to 45.3% across a gestational age span of 57–125 days in seven individuals, suggesting that although *MEG3-IG* methylation is established during gametogenesis, this region may continue to subtly accrue methylation post-fertilization in the brain, at least during the gestational period analyzed (here, ∼0.6% methylation per week). Larger studies will be required to confirm these findings.

### Comparison of DMR Methylation Levels in Umbilical Cord Blood and Buccal Cells

Figure 5 shows comparisons of methylation levels at the two DMRs regulating *IGF2* in matched sets of DNA extracted from umbilical cord blood and buccal cells from nine infants. As shown in Figures 5A and 5B, the *IGF2* DMR shows consistent but slightly lower methylation across buccal cells than in umbilical cord blood, while the *H19* DMR (Figure 5A and 5C) does not exhibit significantly different methylation between these tissues. These results are consistent with the findings shown in Figure 3 for these two DMRs, in which unmatched umbilical cord blood and buccal cell specimens were examined. Furthermore, they show that methylation status at these two DMRs is proportionately similar (*IGF2* DMR; average 7.2% difference; paired t test p = 0.0002) or nearly identical (*H19* DMR; average 0.09% difference; p = 0.9612).

**Figure 6:**
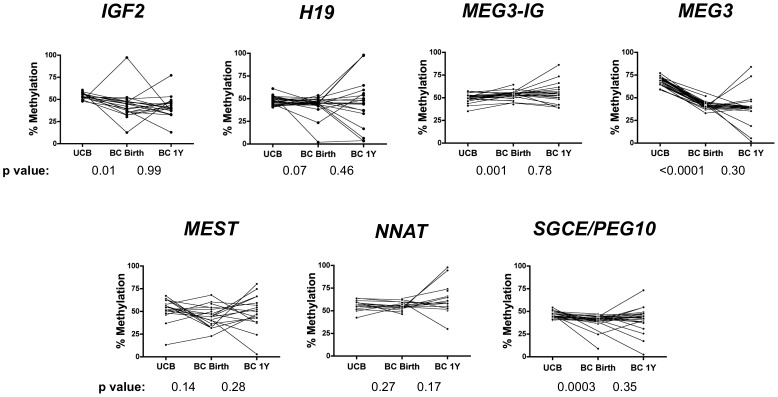
Inter-individual DMR methylation profiles in UCB and buccal cells over time. Analysis of up to 30 matched sets of umbilical cord blood (UCB) specimens, buccal cells taken at birth (BC Birth) and buccal cells taken at one year of age (BC 1Y) at the seven imprinted DMRs. Paired t tests were used to evaluate relationships between UCB and BC Birth as well as BC Birth and BC 1Y. P values are shown below each paired set of data.

We next examined the seven imprinted DMRs in a group of 30 newborns for whom we had DNA from UCB and buccal cells at birth along with buccal cells taken at approximately one year of age. Comparing UCB with buccal cells taken at birth, the *IGF2* and *H19* DMRs again showed the same patterns of methylation as those described above in this independent set of specimens (Figure 6). The *MEST* and *NNAT* DMRs also showed highly similar methylation between buccal cells and UCB. DMRs showing a constant proportionate difference between UCB and buccal cells at birth were the *MEG3-*IG DMR (average 3.0% difference; paired t test p = 0.0003), the *MEG3* DMR (average 27.5% difference; paired t test p<0.0001) and the *SGCE/PEG10* DMR (average 5.7% difference; paired t test p = 0.002).

### Stability of Methylation Marks between Birth and Age One Year

**Figure 7:**
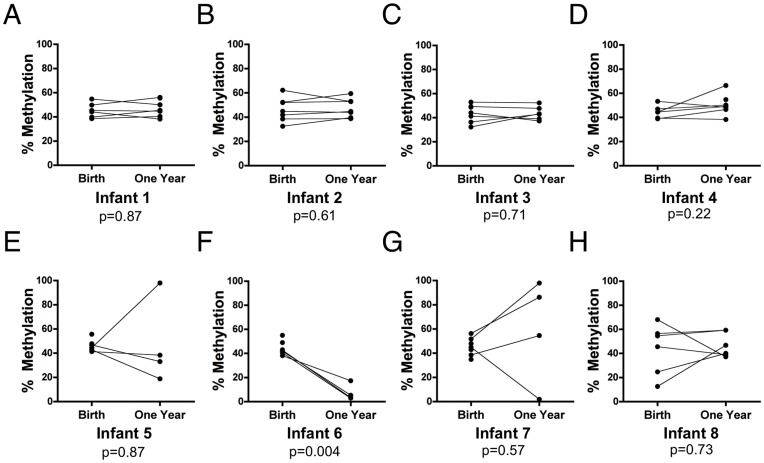
Intra-individual DMR methylation profiles in buccal cells over time. Matched buccal cell specimens from birth and one year of age (a subset of data from Figure 6), showing representative individuals with normal methylation profiles at both time points (panels A–D, infants 1–4) and those with normal methylation at birth that was abnormal for >1 DMR at one year of age (panels E–G, infants 5–8) as well as one individual with an abnormal methylation profile for 3 DMRs at birth that were normal by age one (infant 8). Paired t tests p values are shown for each infant. See Table S3 for methylation values by DMR.

Most infants showed remarkable stability in methylation profiles at these DMRs between birth and one year of age, with no significant group differences in methylation levels for these two points in time (Figure 6). However, there were notable changes in several infants at some DMRs, suggesting methylation at these DMRs may represent a more global disruption in DMR methylation in these children, as many of the same individuals were affected. While most had relatively normal methylation profiles in buccal cells both at birth and at one year of age (Figure 7A–7C; data are shown for a subset of the individuals in Figure 6), some infants showed evidence of sporadic changes at one year of age from a normal methylation profile at birth (e.g., Figure 7D). In contrast, there were several individuals who exhibited wide shifts in methylation at multiple DMRs between these two time points (Figure 7E–7G). For these infants, methylation profiles appear to be relatively normal at birth, but show substantial shifts at multiple DMRs by one year of age. Another infant born at 30 weeks gestation (Figure 7H) exhibited abnormal DMR methylation at birth that seemed to return to a more normal (∼50%) pattern by one year of age (see Table S3 for methylation values by DMR). The reason(s) for these shifts cannot be conclusively determined due to small sample size and the observational design of the study, but raise the possibility that methylation at imprinted gene DMRs may be even more malleable than previously appreciated, at least in this tissue type, which is immediately proximal to a wealth of airborne and food-related environmental stimuli.

### Major Cord Blood Fractions Exhibit Similar Patterns of DMR Methylation

**Figure 8:**
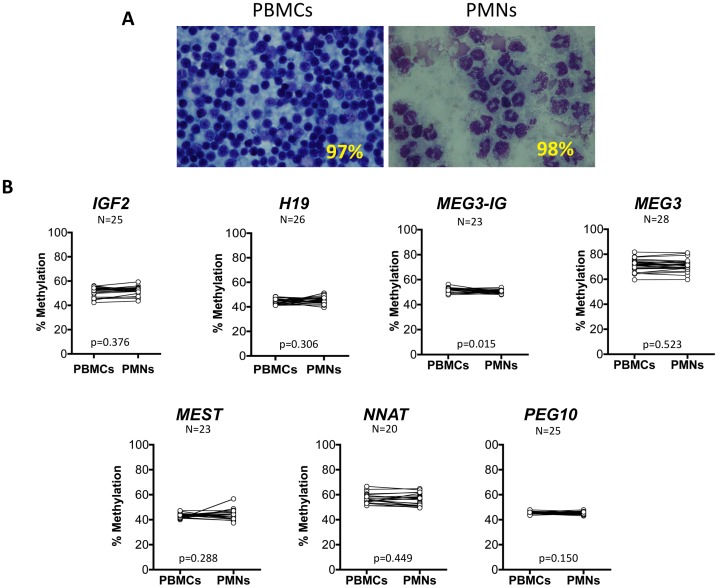
Methylation at imprinted DMRs in the major umbilical cord blood fractions. (A) Representative Giemsa staining of peripheral blood monocytic cells (PBMCs) and polymorphonuclear cells (PMNs) from umbilical cord blood showing percent purity. (B) Methylation at the seven imprinted gene DMRs analyzed in this study did not significantly differ between blood fractions from up to 28 paired specimens analyzed based on paired t tests, except at the *MEG3-IG* DMR, for which the difference in mean methylation between fractions was 1.14%.

The multiple cell types in human umbilical cord blood may have different epigenetic profiles that could complicate interpretation of methylation findings. We analyzed up to 28 UCB specimens to determine if the major cell fractions, polymorphonuclear cells (PMNs) and mononuclear cells (PBMCs), share similar methylation profiles at the seven imprinted DMRs. Fraction purity was determined by counting the PMNs and PBMCs present in Giemsa stained specimens (representative data shown in Figure 8A). As shown in Figure 8B, the DMR methylation profiles in these matched cell fractions are indistinguishable as analyzed by paired t tests, except for the *MEG3-IG* DMR, which showed a mean difference of 1.14% between fractions (p = 0.015). These results support use of unfractionated UCB for analysis of the DMRs associated with these genes.

## Discussion

Although epigenetic mechanisms have been proposed to mediate the association between early exposures and common chronic adult-onset diseases as adaptive responses, a major impediment has been the lack of suitable epigenetic biomarkers to detect historical (periconceptional, prenatal and early postnatal) alterations in the epigenome that are stable and able to be assessed through analysis of accessible tissues from otherwise healthy human subjects. Herein we have analyzed the temporal stability of DNA methylation at imprinted gene DMRs and our results suggest that most of these regions may be suitable for evaluation in retrospective studies, the most efficient design to study common chronic diseases.

We examined methylation at seven imprinted gene DMRs using bisulfite pyrosequencing. This technique is considered one of the best for loci-specific DNA methylation analysis [39], [40], [41] since it allows for single nucleotide resolution of DNA methylation profiles while simultaneously providing a quantitative measure of the level of methylation at each CpG dinucleotide throughout the target sequence. Among our key findings was that DNA methylation levels are equivalent at the *H19*, *MEST* and *SGCE/PEG10* DMRs in eleven analyzed tissue types derived from the three germ layers at 58 to 125 days gestation. Furthermore, methylation levels at these DMRs were also similar between buccal cells and umbilical cord blood at birth and between birth and age one year. At the *IGF2, MEG3* and *NNAT* DMRs, methylation differences were also similar in all tissues except the brain, kidney (*IGF2*), liver, thymus (*MEG3*) and placenta. Intriguingly, postnatal methylation levels at birth and at age one year were similar for *IGF2* and both *MEG3* DMRs while methylation differences between birth and age one year were evident at DMRs regulating *H19*, *MEST* and *NNAT*. These data are consistent with the interpretation that methylation at some DMRs is stable while others exhibit more malleability *in utero* and early in the postnatal period.

Validation of pyrosequencing assays for imprinted DMRs is challenging because of the inherent ∼50% baseline level of methylation present in biological specimens. This makes it difficult to test for assay performance in the <50% methylation range. Therefore, we used two approaches for assay validation, including plasmids containing cloned inserts with the bisulfite modified versions of the completely methylated or unmethylated sequences for the *IGF2* and *H19* DMRs, and commercially available whole genome amplified DNAs for the remaining DMRs. Whole genome amplification effectively “erases” cytosine methylation since DNA methyltransferase enzymes are not present to restore post-synthesis methylation to the nascent amplicons. Following amplification, a portion of the product, destined to become the fully methylated DNA, is treated with the bacterial *M.Sss*I methyltransferase enzyme, which methylates CpG dinucleotides. The unmethylated and methylated whole genome amplification products are then treated with sodium bisulfite to generate the templates that can be used for validation and as controls in methylation analysis experiments. In our experience, the commercially available whole genome amplified methylated DNA often exhibits locus-dependent incomplete methylation, with methylation levels ranging from ∼80%–95% (e.g., see Figure 1). Validation of assay performance involves analysis of defined mixtures of the unmethylated and methylated templates in order to show a linear signal of detection across the dynamic range of the assay. Deviations from unity indicate potential bias in amplification. Such a result is of concern and indicates a need for assay redesign if the intent is to derive absolute quantification of methylation levels at CpG dinucleotides. On the other hand, if the intent is to demonstrate methylation differences between individual specimens or groups of specimens, then bias in one or the other direction may not be as critical as being able to demonstrate that there are relative differences in methylation that can be detected.

We used an alternative strategy, first published by Wong et al. [35], to validate assay performance for the *IGF2* and *H19* DMRs, and more specifically, to address the ability to detect small differences in methylation over the dynamic range of the assays. This approach relied on the analysis of plasmids containing bisulfite modified sequence inserts representing the fully methylated and fully unmethylated versions of the target sequence. The source of the plasmid inserts was normal human lymphocyte genomic DNA that had been bisulfite modified, amplified, ligated into pGEM T-Easy vectors, transformed, screened and sequenced to identify clones containing the desired methylation profiles. The bacterial clones were then grown at “midi-prep” scale to generate sufficient amounts of plasmid DNA and to improve accuracy of the detection of plasmid concentrations – both critical to generating mixtures of narrowly-defined ratios. The results for both DMRs analyzed show that 5% differences in methylation are distinguished by pyrosequencing over the entirety of the possible range of values and support the power of this technique for quantitative methylation analysis of human populations.

Methylation profiles at most of the imprinted gene DMRs analyzed showed little variability across a broad range of prenatal and postnatal tissues from different individuals. They also show a baseline level of methylation that is close to the theoretical 50% level of methylation expected for regions whose methylation status is differentially established on parental alleles. Unlike other regions of the genome that show tissue-specific methylation or that are unmethylated, normal imprinted gene DMR methylation is at a level that is approximately equidistant from the two possible extreme values. Given that we found this profile is a homogeneous feature of multiple tissues in humans, subtle endogenously- or exogenously-induced deviations that occur during early development would be readily detected in available tissues and can provide information regarding influential forces that led to such deviation. These findings leave us well-positioned to ask questions about the identity and nature of such influential prenatal forces that are capable of shifting imprinted gene DMR methylation profiles, as we have already demonstrated for *in utero* exposures to maternal cigarette smoking, folic acid and antidepressants [32], [42], [43] and others have demonstrated in relation to exposure to famine conditions [25], [30]. Such deviations in DMR methylation will also very likely be of functional importance since imprinted genes play important roles in such fundamental processes as cellular differentiation [44], [45], [46], [47], prenatal and postnatal growth [48], [49], neurological function and memory [50], [51], insulin pathway function [52], [53], [54], [55], nurturing behaviors [56], [57] and when deregulated, in cancer [58].

A previous study by Talens et al. [59] showed no significant differences between the major cell fractions in peripheral blood at the imprinted loci analyzed, consistent with our results in umbilical cord blood. They also reported somewhat variable methylation between individuals at loci not exhibiting extremes of methylation patterns, strong correlations between methylation profiles in blood and matched buccal cells and that methylation patterns were stable over time at most imprinted loci using buccal cell specimens. While the interval between buccal cell sampling in the Talens et al. study was up to 11 years with a baseline of 14–62 years of age, our study focused on methylation profiles across tissues before birth, at the time of birth and again at one year of age to determine the level of methylation present in tissues that are naïve to the postnatal environment. We therefore compared DMR methylation in umbilical cord blood to that obtained from buccal cells in matched samples. Three of the DMRs analyzed showed a significant but proportionate difference in the level of methylation detected in these two cell types while the remaining four DMRs showed no significant differences. The more limited amounts of DNA available from buccal cell specimens makes use of umbilical cord blood more desirable, but caution must be used in interpreting the results for DMRs exhibiting differences between tissue types and adjustments made to account for such predefined proportionate differences.

We also compared DMR methylation profile in buccal cell specimens taken at birth to those taken from the same individuals when they reached one year of age. Most DMR methylation profiles were within a normal range (defined here for purposes of comparison as the percent methylation range that includes the average level of methylation detected across all examined tissues plus and minus one standard deviation) at both time points, but there were specimens that exhibited variant methylation at these DMRs. We found that subset of the individuals analyzed appeared to have deregulation of multiple DMRs, suggesting a more general epigenetic disruption may exist in these individuals. Intriguingly, several individuals showed a shift over the first year of postnatal life from a normal to abnormal methylation profile, and one individual showed an abnormal methylation profile for multiple DMRs at birth that largely returned to normal at age one. These results contrast with findings from a recent survey of imprinted DMRs in adults [60] in which DMR stability was maintained across tissues. Although these findings require replication in larger studies, they suggest that methylation at imprinted DMRs is malleable early in life.

A potential limitation of our study is the small numbers of samples evaluated; however, these provide the first evidence for the stability of DNA methylation during prenatal and early postnatal life in humans. Our study also provides methods by which future epidemiologic studies might validate the stability of epigenetic marks proposed for evaluation in retrospectively collected exposure data. Another limitation is that our data is restricted to analysis of DNA methylation marks as measured during prenatal and early postnatal development. We are currently following our Newborn Epigenetics STudy infants [33] in early childhood to determine whether DMR methylation marks are malleable beyond the first year of life. We cannot exclude the possibility that some interindividual differences in DMR methylation are inherited as opposed to a *de novo* event in the current generation. However, this is unlikely, since epigenetic reprogramming during gametogenesis normally results in the erasure of the methylation at imprinted DMRs from the prior generation and establishment of new methylation profiles that reflect the sex of the individual harboring the gametes. Finally, single nucleotide polymorphisms or copy number variants in genes that affect DNA methylation, or those proximal to (or within) the sites of DNA methylation, can also exert a powerful influence on DNA methylation profiles [61], [62], [63] that may drive interindividual methylation differences but were not assessed in this.study.

In summary, we have shown comparable DMR methylation levels at birth between two tissues – umbilical cord blood and buccal cells. DMR methylation levels were also similar between birth and one year of age at three independent imprinted gene DMRs. At these same DMRs, methylation levels were also comparable across multiple tissue types from human conceptuses aged 58–125 days gestation and between the major cell fractions in umbilical cord blood. Although small sizes limit inference, this study provides support for the potential utility of some of these DMRs as early exposure assessment tools for use in epidemiological studies.

## Supporting Information

Table S1 **Human conceptual tissue specimens and DMRs analyzed.**
(DOCX)

Table S2 **Correlations between gestational age and DMR methylation.**
(DOCX)

Table S3 **DMR methylation levels in buccal cells at birth and buccal cells at one year of age in 8 infants.**
(DOCX)
