# Supplementary material for: Differentially Methylated Regions of Imprinted Genes in Prenatal, Perinatal and Postnatal Human Tissues
Source: PLoS One. 2012 Jul 13;7(7):e40924. doi: 10.1371/journal.pone.0040924 (PMC3396645; doi:10.1371/journal.pone.0040924)
Supplement: Table S1 — Human conceptual tissue specimens and DMRs analyzed. (DOCX) [file pone.0040924.s001.docx]

**Table S1. Specimens and DMRs analyzed**

| **Number** | **Specimen** | **Sex** | **Days**  **Gestation** | ***PEG10*** | ***IGF2*** | ***H19*** | ***MEST*** | ***MEG3-IG*** | ***MEG3*** | ***NNAT*** |
| --- | --- | --- | --- | --- | --- | --- | --- | --- | --- | --- |
| 1 | Adrenal | F | 57 | ✓ | ✓ | ✓ | ✓ | ✓ | ✓ | ✓ |
|  | Brain |  |  |  | ✓ | ✓ | ✓ | ✓ | ✓ | ✓ |
|  | Decidua |  |  |  | ✓ | ✓ |  | ✓ | ✓ | ✓ |
|  | Intestine |  |  | ✓ | ✓ | ✓ | ✓ | ✓ | ✓ | ✓ |
|  | Kidney |  |  |  | ✓ | ✓ | ✓ | ✓ | ✓ | ✓ |
|  | Liver |  |  | ✓ | ✓ | ✓ | ✓ | ✓ | ✓ | ✓ |
|  | Placenta |  |  |  | ✓ | ✓ | ✓ | ✓ | ✓ | ✓ |
|  | U. Cord |  |  | ✓ | ✓ | ✓ | ✓ | ✓ | ✓ | ✓ |
| 2 | Adrenal | M | 58 | ✓ | ✓ | ✓ | ✓ | ✓ | ✓ | ✓ |
|  | Brain |  |  |  | ✓ | ✓ |  |  |  |  |
|  | Decidua |  |  |  | ✓ | ✓ | ✓ | ✓ | ✓ | ✓ |
|  | Eye |  |  | ✓ | ✓ | ✓ | ✓ | ✓ | ✓ | ✓ |
|  | Lung |  |  | ✓ | ✓ | ✓ | ✓ | ✓ | ✓ | ✓ |
|  | Placenta |  |  |  | ✓ | ✓ | ✓ | ✓ | ✓ | ✓ |
|  | U. Cord |  |  |  | ✓ | ✓ | ✓ | ✓ | ✓ | ✓ |
| 3 | Adrenal | M | 80 |  | ✓ | ✓ |  |  |  |  |
|  | Brain |  |  | ✓ | ✓ | ✓ | ✓ | ✓ | ✓ | ✓ |
|  | Heart |  |  |  | ✓ | ✓ |  |  |  |  |
|  | Intestine |  |  |  |  | ✓ |  |  |  |  |
|  | Kidney |  |  | ✓ | ✓ | ✓ | ✓ | ✓ | ✓ | ✓ |
|  | Liver |  |  | ✓ | ✓ | ✓ | ✓ | ✓ | ✓ | ✓ |
|  | Lung |  |  |  | ✓ | ✓ |  |  |  |  |
|  | Pancreas |  |  |  | ✓ | ✓ |  |  |  |  |
|  | Placenta |  |  |  | ✓ | ✓ |  |  |  |  |
| 4 | Brain | F | 80 |  | ✓ |  |  |  |  |  |
|  | Liver |  |  |  | ✓ |  |  |  |  |  |
|  | Kidney |  |  |  | ✓ |  |  |  |  |  |
| 5 | Decidua | F | 87 |  | ✓ | ✓ |  |  |  |  |
|  | Heart |  |  |  | ✓ | ✓ |  |  |  |  |
|  | Intestine |  |  |  | ✓ | ✓ |  |  |  |  |
|  | Kidney |  |  |  | ✓ | ✓ |  |  |  |  |
|  | Liver |  |  |  | ✓ | ✓ |  |  |  |  |
|  | Lung |  |  |  | ✓ | ✓ |  |  |  |  |
|  | Muscle |  |  |  | ✓ | ✓ |  |  |  |  |
|  | Pancreas |  |  |  | ✓ | ✓ |  |  |  |  |
|  | Thymus |  |  |  | ✓ | ✓ |  |  |  |  |
| 6 | Adrenal | F | 94 |  | ✓ | ✓ |  |  |  |  |
|  | Brain |  |  |  | ✓ | ✓ |  |  |  |  |
|  | Decidua |  |  |  | ✓ | ✓ |  |  |  |  |
|  | Heart |  |  |  | ✓ | ✓ |  |  |  |  |
|  | Intestine |  |  |  | ✓ | ✓ |  |  |  |  |
|  | Kidney |  |  |  | ✓ | ✓ |  |  |  |  |
|  | Liver |  |  |  |  | ✓ |  |  |  |  |
|  | Lung |  |  |  | ✓ | ✓ |  |  |  |  |
|  | Muscle |  |  |  | ✓ | ✓ |  |  |  |  |
|  | Pancreas |  |  |  | ✓ |  |  |  |  |  |
|  | Placenta |  |  |  | ✓ | ✓ |  |  |  |  |
|  | Spleen |  |  |  | ✓ | ✓ |  |  |  |  |
|  | Thymus |  |  |  | ✓ | ✓ |  |  |  |  |
| 7 | Brain | M | 98 |  |  | ✓ |  |  |  |  |
|  | Kidney |  |  |  |  | ✓ |  |  |  |  |
|  | Liver |  |  |  |  | ✓ |  |  |  |  |
| 8 | Adrenal | F | 101 |  | ✓ |  |  |  |  |  |
|  | Brain |  |  |  | ✓ | ✓ |  |  |  |  |
|  | Decidua |  |  |  |  | ✓ |  |  |  |  |
|  | Heart |  |  |  | ✓ | ✓ |  |  |  |  |
|  | Intestine |  |  |  | ✓ | ✓ |  |  |  |  |
|  | Kidney |  |  |  | ✓ | ✓ |  |  |  |  |
|  | Placenta |  |  |  | ✓ | ✓ |  |  |  |  |
|  | Thymus |  |  |  |  | ✓ |  |  |  |  |
|  | U. Cord |  |  |  | ✓ |  |  |  |  |  |
| 9 | Adrenal | F | 105 |  | ✓ | ✓ |  |  |  |  |
|  | Brain |  |  | ✓ | ✓ | ✓ | ✓ | ✓ | ✓ | ✓ |
|  | Decidua |  |  |  |  | ✓ |  |  |  |  |
|  | Heart |  |  |  | ✓ | ✓ |  |  |  |  |
|  | Intestine |  |  |  | ✓ | ✓ |  |  |  |  |
|  | Kidney |  |  | ✓ | ✓ | ✓ | ✓ | ✓ | ✓ | ✓ |
|  | Liver |  |  | ✓ | ✓ | ✓ | ✓ | ✓ | ✓ |  |
|  | Lung |  |  |  | ✓ | ✓ |  |  |  |  |
|  | Muscle |  |  |  | ✓ | ✓ |  |  |  |  |
|  | Pancreas |  |  |  | ✓ | ✓ |  |  |  |  |
|  | Spleen |  |  |  | ✓ | ✓ |  |  |  |  |
|  | Thymus |  |  |  | ✓ | ✓ |  |  |  |  |
|  | U. Cord |  |  |  | ✓ | ✓ |  |  |  |  |
| 10 | Adrenal | F | 108 |  |  | ✓ |  |  |  |  |
|  | Brain |  |  | ✓ |  | ✓ | ✓ | ✓ | ✓ | ✓ |
|  | Decidua |  |  |  |  | ✓ |  |  |  |  |
|  | Gonad |  |  |  |  | ✓ |  |  |  |  |
|  | Heart |  |  |  |  | ✓ |  |  |  |  |
|  | Intestine |  |  |  |  | ✓ |  |  |  |  |
|  | Kidney |  |  | ✓ |  | ✓ | ✓ | ✓ | ✓ | ✓ |
|  | Liver |  |  | ✓ |  | ✓ | ✓ | ✓ |  |  |
|  | Lung |  |  |  |  | ✓ |  |  |  |  |
|  | Muscle |  |  |  |  | ✓ |  |  |  |  |
|  | Pancreas |  |  |  |  | ✓ |  |  |  |  |
|  | Placenta |  |  |  |  | ✓ |  |  |  |  |
|  | U. Cord |  |  |  |  | ✓ |  |  |  |  |
| 11 | Adrenal | F | 108 |  | ✓ | ✓ |  |  |  |  |
|  | Brain |  |  | ✓ | ✓ | ✓ | ✓ | ✓ | ✓ | ✓ |
|  | Decidua |  |  |  | ✓ | ✓ |  |  |  |  |
|  | Heart |  |  |  | ✓ | ✓ |  |  |  |  |
|  | Intestine |  |  |  | ✓ | ✓ |  |  |  |  |
|  | Kidney |  |  | ✓ | ✓ | ✓ | ✓ | ✓ | ✓ | ✓ |
|  | Liver |  |  | ✓ | ✓ | ✓ | ✓ | ✓ | ✓ | ✓ |
|  | Muscle |  |  |  | ✓ | ✓ |  |  |  |  |
|  | Pancreas |  |  |  | ✓ | ✓ |  |  |  |  |
|  | Placenta |  |  |  | ✓ | ✓ |  |  |  |  |
|  | Spleen |  |  |  | ✓ | ✓ |  |  |  |  |
|  | Thymus |  |  |  | ✓ | ✓ |  |  |  |  |
|  | U. Cord |  |  |  | ✓ | ✓ |  |  |  |  |
| 12 | Brain | M | 113 |  | ✓ |  |  |  |  |  |
|  | Kidney |  |  |  | ✓ |  |  |  |  |  |
|  | Liver |  |  |  | ✓ |  |  |  |  |  |
| 13 | Brain | M | 120 |  |  | ✓ |  |  |  |  |
|  | Kidney |  |  |  |  | ✓ |  |  |  |  |
|  | Liver |  |  |  |  | ✓ |  |  |  |  |
| 14 | Brain | F | 120 |  |  | ✓ |  |  |  |  |
|  | Kidney |  |  |  |  | ✓ |  |  |  |  |
|  | Liver |  |  |  |  | ✓ |  |  |  |  |
| 15 | Adrenal | F | 122 | ✓ | ✓ | ✓ |  |  | ✓ | ✓ |
|  | Brain |  |  | ✓ | ✓ | ✓ |  | ✓ | ✓ |  |
|  | Decidua |  |  | ✓ | ✓ | ✓ |  | ✓ | ✓ | ✓ |
|  | Gonad |  |  | ✓ | ✓ | ✓ | ✓ | ✓ | ✓ | ✓ |
|  | Heart |  |  | ✓ | ✓ | ✓ | ✓ | ✓ | ✓ | ✓ |
|  | Intestine |  |  | ✓ | ✓ | ✓ | ✓ | ✓ | ✓ | ✓ |
|  | Kidney |  |  |  | ✓ | ✓ | ✓ | ✓ | ✓ |  |
|  | Liver |  |  |  | ✓ | ✓ |  | ✓ | ✓ |  |
|  | Lung |  |  | ✓ | ✓ | ✓ | ✓ | ✓ | ✓ | ✓ |
|  | Muscle |  |  | ✓ | ✓ | ✓ | ✓ |  |  | ✓ |
|  | Pancreas |  |  | ✓ | ✓ | ✓ |  | ✓ | ✓ | ✓ |
|  | Placenta |  |  |  | ✓ | ✓ | ✓ | ✓ | ✓ |  |
|  | Thymus |  |  |  | ✓ | ✓ | ✓ | ✓ | ✓ | ✓ |
|  | U. Cord |  |  |  | ✓ | ✓ | ✓ | ✓ | ✓ | ✓ |
| 16 | Adrenal | M | 125 | ✓ | ✓ | ✓ |  | ✓ | ✓ |  |
|  | Brain |  |  | ✓ | ✓ | ✓ | ✓ | ✓ | ✓ | ✓ |
|  | Decidua |  |  | ✓ | ✓ | ✓ | ✓ | ✓ | ✓ | ✓ |
|  | Heart |  |  | ✓ | ✓ | ✓ | ✓ | ✓ | ✓ | ✓ |
|  | Intestine |  |  | ✓ | ✓ | ✓ |  | ✓ | ✓ | ✓ |
|  | Liver |  |  | ✓ | ✓ | ✓ | ✓ | ✓ | ✓ | ✓ |
|  | Lung |  |  | ✓ | ✓ | ✓ | ✓ | ✓ | ✓ | ✓ |
|  | Pancreas |  |  | ✓ | ✓ | ✓ |  | ✓ | ✓ |  |
|  | Placenta |  |  | ✓ | ✓ | ✓ | ✓ | ✓ | ✓ | ✓ |
|  | Spleen |  |  | ✓ | ✓ | ✓ |  | ✓ | ✓ | ✓ |
|  | Thymus |  |  | ✓ | ✓ | ✓ |  |  | ✓ | ✓ |
|  | U. Cord |  |  | ✓ | ✓ | ✓ | ✓ | ✓ | ✓ | ✓ |
