# Supplementary material for: Differentially Methylated Regions of Imprinted Genes in Prenatal, Perinatal and Postnatal Human Tissues
Source: PLoS One. 2012 Jul 13;7(7):e40924. doi: 10.1371/journal.pone.0040924 (PMC3396645; doi:10.1371/journal.pone.0040924)
Supplement: Table S2 — Correlations between gestational age and DMR methylation. (DOCX) [file pone.0040924.s002.docx]

|  | ***IGF2*** | ***H19*** | ***MEG3*** | ***MEG3-IG*** | ***NNAT*** | ***MEST*** | ***PEG10/SGCE*** |
| --- | --- | --- | --- | --- | --- | --- | --- |
|  | R | R | R | R | R | R | R |
|  | (p) | (p) | (p) | (p) | (p) | (p) | (p) |
| Adrenal | 0.53 | -0.13 | - | - | - | - | - |
|  | (0.53) | (0.74) | - | - | - | - | - |
| Brain | -0.41 | -0.03 | 0.32 | **0.90** | 0.09 | 0.12 | 0.12 |
|  | (0.21) | (0.93) | (0.44) | **(0.01)** | (0.92) | (0.80) | (0.80) |
| Decidua | -0.11 | 0.27 | - | - | - | - | - |
|  | (0.84) | (0.45) | - | - | - | - | - |
| Heart | -0.26 | -0.51 | - | - | - | - | - |
|  | (0.54) | (0.16) | - | - | - | - | - |
| Intestine | -0.14 | -0.54 | - | - | - | - | - |
|  | (0.75) | (0.11) | - | - | - | - | - |
| Kidney | 0.30 | -0.23 | -0.49 | 0.49 | 0.66 | 0.01 | - |
|  | (0.41) | (0.47) | (0.36) | (0.36) | (0.23) | (1.0) | - |
| Liver | 0.09 | -0.05 | 0.90 | 0.61 | - | -0.03 | 0.26 |
|  | (0.81) | (0.89) | (0.08) | (0.17) | - | (1.0) | (0.66) |
| Lung | 0.29 | -0.14 | - | - | - | - | - |
|  | (0.56) | (0.75) | - | - | - | - | - |
| Muscle | -0.80 | -0.41 | - | - | - | - | - |
|  | (0.13) | (0.42) | - | - | - | - | - |
| Pancreas | 0.21 | -0.14 | - | - | - | - | - |
|  | (0.66) | (0.78) | - | - | - | - | - |
| Placenta | 0.38 | -0.05 | - | - | - | - | - |
|  | (0.34) | (0.91) | - | - | - | - | - |
| Thymus | -0.49 | -0.39 | - | - | - | - | - |
|  | (0.36) | (0.40) | - | - | - | - | - |
| Umbilical Cord | 0.11 | -0.40 | - | - | - | - | - |
|  | (0.84) | (0.40) | - | - | - | - | - |

**Table S2.** Correlations between gestational age and methylation*

* Correlation analysis was performed only for tissues in which there were ≥5 specimens with methylation values
