# Supplementary material for: Differentially Methylated Regions of Imprinted Genes in Prenatal, Perinatal and Postnatal Human Tissues
Source: PLoS One. 2012 Jul 13;7(7):e40924. doi: 10.1371/journal.pone.0040924 (PMC3396645; doi:10.1371/journal.pone.0040924)
Supplement: Table S3 — DMR methylation levels in buccal cells at birth and buccal cells at one year of age in 8 infants. (DOCX) [file pone.0040924.s003.docx]

|  | *IGF2* | | *H19* | | *MEG3-IG* | | *MEG3* | | *MEST* | | *NNAT* | | *PEG10* | |
| --- | --- | --- | --- | --- | --- | --- | --- | --- | --- | --- | --- | --- | --- | --- |
|  | Birth | 1 year | Birth | 1 year | Birth | 1 year | Birth | 1 year | Birth | 1 year | Birth | 1 year | Birth | 1 year |
| Infant 1 | 39.9 | 45.6 | 45.4 | 44.7 | 49.8 | 56.2 | 44.2 | 38.2 | 54.8 | 50.0 | - | 55.5 | 38.7 | 40.4 |
| Infant 2 | 32.5 | 40.0 | 44.9 | 43.9 | 52.1 | 53.1 | 38.5 | 38.9 | 52.3 | 53.1 | 62.2 | 52.8 | 41.8 | 44.7 |
| Infant 3 | 32.3 | 43.3 | 49.3 | 47.8 | 52.9 | 52.3 | 41.3 | 39.1 | 36.4 | 43.0 | 48.7 | - | 44.1 | 37.3 |
| Infant 4 | 38.9 | 46.5 | 46.9 | 50.3 | 53.4 | 48.4 | 39.5 | 38.3 | 44.4 | 66.4 | - | 54.8 | 44.4 | 49.2 |
| Infant 5 | 46.9 | 33.1 | 44.5 | 98.1 | 46.9 | - | 42.9 | 18.9 | 47.8 | - | 55.7 | - | 41.4 | 38.4 |
| Infant 6 | 43.1 | - | 42.8 | 3.4 | 49.0 | - | 42.0 | 5.4 | 40.7 | 2.9 | 55.1 | - | 38.2 | 17.4 |
| Infant 7 | 48.0 | - | 43.0 | - | 56.3 | 86.3 | 44.9 | 2.0 | 34.9 | - | 51.8 | 98.0 | 38.6 | 54.7 |
| Infant 8 | 12.6 | 46.8 | - | - | 54.6 | 59.3 | 45.7 | 39.0 | 68.1 | 37.2 | 56.4 | 59.3 | 24.7 | 40.0 |

**Table S3.** Methylation levels in buccal cells*

* Values match Figure 6 in the main manuscript
